# Safer tattooing interventions in prisons: a systematic review and call to action

**DOI:** 10.1186/s12889-018-5867-x

**Published:** 2018-08-15

**Authors:** Nguyen Toan Tran, Célestine Dubost, Stéphanie Baggio, Laurent Gétaz, Hans Wolff

**Affiliations:** 1 Division of Health in Prison, Geneva University Hospitals and University of Geneva, Ch. du Petit-Bel-Air 2, CH-1225 Chêne-Bourg, Switzerland; 2 Australian Centre for Public and Population Health Research, Faculty of Health, University of Technology, PO Box 123, Sydney, NSW 2007 Australia

**Keywords:** Safer tattooing, Harm reduction, Detention, Prison, Human rights, Call to action, Stepwise approach

## Abstract

**Background:**

Worldwide more than ten million people are detained at any given time. Between 5 and 60% of people experiencing incarceration report receipt of a tattoo in prison – mostly clandestine, which is associated with risks of blood-borne infections (BBIs). Although safer tattooing techniques are effective in preventing BBI transmission and available to the general population, there is limited knowledge about the impact of safer tattooing strategies in prisons in terms of health outcomes, changes in knowledge and behaviors, and best practice models for implementation. The objective of this research was to identify and review safer tattooing interventions.

**Methods:**

We conducted a systematic review of the literature. Studies of all design types were included if they were published until 27 June 2018, the population was incarcerated adults, they reported quantitative outcomes, and were published in English, French, or Spanish.

**Results:**

Of 55 papers retrieved from the initial search, no peer-reviewed article was identified. One paper from the grey literature described a multi-site pilot project in Canada. Its evaluation suggested that the project was effective in enhancing knowledge of incarcerated people and prison staff on standard precautions, had the potential to reduce harm, provided vocational opportunities, and was feasible although enhancements were needed to improve implementation issues and efficiency.

**Conclusions:**

Although access to preventive services, including to safer tattooing interventions, is a human right and recommended by United Nations agencies as part of a comprehensive package of harm reduction interventions in prisons, this review identified only a few promising strategies for safer tattooing interventions in carceral settings. We call upon governments, criminal justice authorities, non-governmental organizations, and academic institutions to implement safer tattooing projects that adhere to the following guiding principles: i) integration of methodologically-rigorous implementation research; ii) involvement of key stakeholders (incarcerated people, prison authorities, research partners) in the project design, implementation, and research; iii) integration into a comprehensive package of BBI prevention, treatment, and care, using a stepwise approach that considers local resources and acceptability; and iv) publication and dissemination of findings, and scaling up efforts.

**Prospero Registration:**

CRD42017072502.

**Electronic supplementary material:**

The online version of this article (10.1186/s12889-018-5867-x) contains supplementary material, which is available to authorized users.

## Background

Worldwide more than ten million people are held in penal institutions [1]. Getting tattoos while in detention is reported to be a common practice among both men and women: 19% of men and nearly 9% of women surveyed in 17 State prisons in Illinois (USA) [2]; 37% of men and 4% of women in seven detention centers in Quebec province, Canada [3]; and in Australia, 28% of men and 27% of women in the five largest State facilities in Victoria, and 25% of men and 13% of women in Queensland [4, 5] . Sex-aggregated results from other studies in carceral settings showed a prevalence of nearly 60% in Puerto Rico [6], 25% in Fiji [7], 26% in Russia [8], 14% in Hungary [9], 11% in England and Wales [10], and 10 to 28% in Scotland [11]. Results in men-only facilities indicated a prevalence of 5% in Iran [12], 11 to 18% in Bosnia and Herzegovina [13, 14], and 28% in Lesotho [15]. In a cross-sectional survey in six prisons in Europe (France, Germany, Italy, the Netherlands, Scotland, and Sweden), the prevalence of in-prison tattooing ranged from 6 to 43%, with a total prevalence of 18% [16]. People who were tattooed in prison reported infrequent single use of tattoo equipment (8–37%). If cleaning took place, it was reportedly done with water and/or heat. In a large survey involving 4425 participants across Canadian prisons, 13% had a tattoo done in prison and were unsure about equipment safety [17].

Tattooing involves skin piercing and potential blood contact. Unsafe tattooing carries an increased risk of poor health outcomes. In addition to adverse skin problems (e.g., bacterial, viral, and mycotic skin infections, allergic skin reactions or lichenoid formations), unsafe tattooing enhances the transmission risk of blood-borne infections (BBIs), such as hepatitis C (HCV), hepatitis B (HBV), and HIV. This is due to the higher prevalence of BBIs among incarcerated people when compared to the general population (*BBI reservoir*), and to the fact that tattooing is largely prohibited or unregulated in prison settings. Therefore, tattooing is often done in a clandestine and unsafe way by using inappropriate equipment, undertaking ineffective sterilization procedures, and sharing tattoo devices (*BBI transmission mode*). With regard to the *transmission mode*, prison-improvised tattooing materials can be made by transforming a mechanical pencil or electric toothbrush or shaver into a tattoo gun (or even using hearing aid batteries as a power source [18]), sharpening metallic guitar strings into tattoo needles, making tattoo ink out of soot (e.g., from burning cooking oil in a tin), and “sterilization” can be done by flaming needles or cleaning them with hot water [11]. Illicit tattooing can also pose health and safety risks to prison staff and to the public at large. Contraband of tattoo-related paraphernalia and staff injuries resulting from puncture with sharp objects directly related to tattooing have been reported [19]. In terms of *reservoir*, prisons worldwide are known to be important sites of BBI transmission, especially where there is a convergence of a high prevalence of BBIs with ongoing injecting drug use [20–22]. People who ever injected drug were found to be twice more likely to get tattooed in prison than those who never injected drug [16]. Unsafe tattooing is a known risk factor for BBIs [23–27], and there have been reports of HCV, HBV, and HIV acquisition through unsafe tattooing in prison [3, 6, 28–33]. As individuals leave prison, BBIs acquired in detention can cause ill health and impact their reintegration in the community, while transmission risks are extended to their partners, friends, families, and the public in general [34, 35].

Against this background, United Nations agencies included in 2013 the *prevention of [HIV] transmission through tattooing, piercing and other forms of skin penetration* into the 15 key interventions that form the comprehensive package of HIV prevention, treatment, and care in prisons and other closed settings. The intervention recommends authorities to *implement initiatives aimed at reducing the sharing and reuse of equipment used for tattooing, piercing and other forms of skin penetration, and the related infections* [36].

There is however limited knowledge about safer tattooing strategies in carceral settings and their impact on health outcomes, changes in people’s knowledge and behaviors, and best practice models for implementation. The prevalence of tattooing among people who are incarcerated was recently synthesized in a systematic review [37]. We carried out this study with the objective to systematically review the literature on safer tattooing interventions in prisons and their impact on individuals’ knowledge and behaviours.

## Methods

We followed the PRISMA guidelines for this review and registered it through PROSPERO (protocol number CRD42017072502) [38, 39].

### Search protocol

We established a Population, Interventions, Comparators, Outcomes, Study design (PICOS) question to guide the review [40]. Our PICOS question was: for people experiencing incarceration, have safer tattooing interventions in prison led to improved health outcomes? We first used Summon, a metasearch engine to access multiple search systems. Such federated engine is a useful tool to initially map the literature as it provides a unified access to extensive search databases, including MEDLINE, CINAHL, SCOPUS and Web of Science [41]. We then triangulated the results with a search on MEDLINE and Web of Sciences. We searched for studies published until 27 June 2018 included. The search strings for the different databases focused on the study population and interventions and did not include comparative populations, outcome, geographic location, or study design (see Table 1). The search was limited to title and abstract and focused in primary intention on online journal articles. We used backward snowballing to find additional papers by searching the reference lists of retrieved articles, and forward snowballing to identify new articles by searching those that cited the retrieved papers.

**Table 1:** Search strings and records retrieved from databases

Sources	Search terms	Retrieved
Databases		
Summon	((Abstract:(tattooing)) OR (Abstract:(tattoo))) AND ((Abstract:(safe)) OR (Abstract:(legal))) AND ((Abstract:(prison)) OR (Abstract:(jail)) OR (Abstract:(incarcerat)) OR (Abstract:(inmate)) OR (Abstract:(detaine)) OR (Abstract:(custod)) OR (Abstract:(detention)) OR (Abstract:(crim)) OR (Abstract:(offend)) OR (Abstract:(correctional)) OR (Abstract:(forensic)) OR (Abstract:(penal institution)))	28
MEDLINE	(((((tattoo[Title/Abstract]) OR tattooing[MeSH Terms])) AND ((legal[Title/Abstract]) OR safe[Title/Abstract]))) AND ((((((((((((prison[MeSH Terms]) OR jail[Title/Abstract]) OR incarcerat[Title/Abstract]) OR inmate[Title/Abstract]) OR detaine[Title/Abstract]) OR custod[Title/Abstract]) OR detention[Title/Abstract]) OR crim[Title/Abstract]) OR offend[Title/Abstract]) OR correctional[Title/Abstract]) OR forensic[Title/Abstract]) OR penal institution[Title/Abstract])	6
Web of Science	(tattooing OR tattoo) AND (safe OR legal) AND (prison OR jail OR incarcerat OR inmate OR detaine OR custod OR detention OR crim OR forensic OR offend OR correctional OR penal institution)	19
Hand searching of references (snowballing)		2
Subtotal		55
Minus duplicates		40
Identified for appraisal		1

### Inclusion and exclusion criteria

Inclusion and exclusion criteria are listed in Table 2.

**Table 2:** Inclusion and exclusion criteria

	Included	Excluded
Topic	Safe, legal tattooing program or initiatives in prison	Prevalence studies on tattoo or associated risk factors
Types of paper / data	Quantitative health evaluations of tattooing programs, including experimental and non-experimental designs that report outcome data	Descriptive quantitative papers with no specific interventions or outcomes; purely qualitative data
Settings	Pre-trial detention settings, prisons (post-trial)	Non-detention settings
Types of publications	Papers in peer-reviewed journals, grey literature reporting on project implementation	Letters, editorials, commentaries
Language	English, French, Spanish	Papers published in other languages than English, French or Spanish
Publication date	Until 27 June 2018	

### Quality assessment

To assess the methodological quality of studies, we used the Effective Public Health Practice Project’s (EPHPP) Quality Assessment Tool for quantitative studies [42]. The studies were rated as strong, moderate, or weak in relation to the following criteria: selection bias, study design, confounders, blinding, data collection method, and withdrawals. We gave an overall rating for each study. Studies without any weak rating for any criterion were overall rated as ‘strong’, those with one weak rating as ‘moderate’, those with two or more weak rating as ‘weak’. Two researchers independently assessed the quality of the studies and resolved any discrepancies through discussion.

### Synthesis

We systematically extracted data from the papers into standardized tables regarding the context, method, characteristics, quality, and findings. We anticipated a heterogenous nature of the retrieved papers and planned a textual narrative approach to the analysis as described by Lucas et al. [40]. This involved a commentary approach to the description and comparison of data that was grouped into various categories. These categories (e.g., carceral-led, medical-led interventions) were informed by the literature and examined in relation to health outcomes. Data was then synthesized by combining studies with similar types of interventions and patterns identified across and within articles. However, a single study was identified for appraisal and, therefore, limited comparative categorization and no quantitative meta-analysis were undertaken.

### Unpublished projects

An unpublished safer tattooing project, which was presented during a European conference on health promotion in prisons (Vienna, 2017) [43], currently exists in Luxemburg since April 2017. In June 2017, we conducted an in-depth interview with a nurse working there with the aim to gain insight into their current practice. The interview guide was articulated around the five dimensions of accessibility as defined by Levesque et al.: availability, acceptability, appropriateness, affordability, and approachability [44]. This framework was also applied to structure the research agenda presented in the Discussion. Written notes were taken by a researcher during the face-to-face interview, for which informed consent was obtained, and transcribed into Word (see English translation from French in Additional file 1). We report key findings in the results.

Another safer tattooing project was piloted in 2010–2011 in Catalonia (Spain) before it was discontinued. We received no answer from the project team and therefore could not conduct in-depth interviews.

## Results

### Search results

**Fig. 1:**
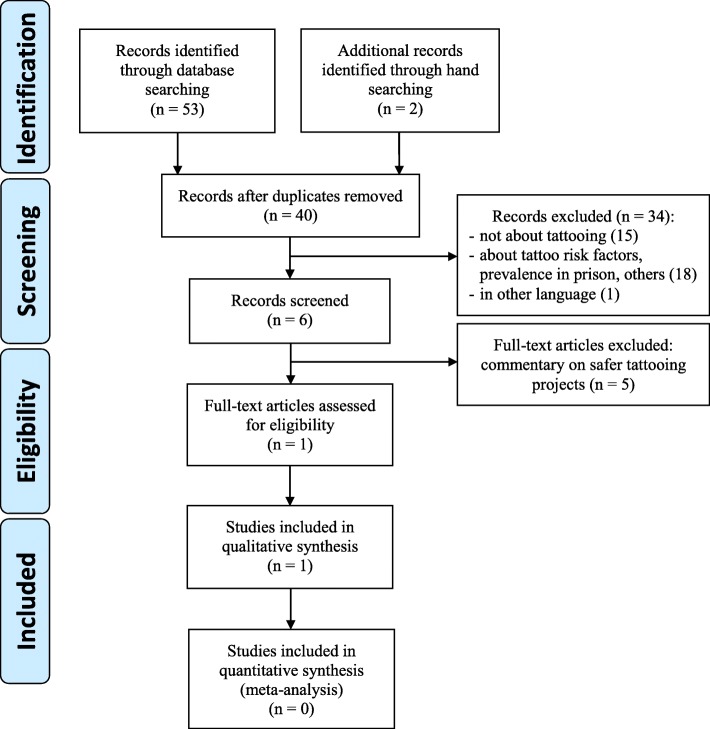
PRISMA 2008 Flow Diagram

The search retrieved 53 publications through database searching of online journal articles, and a further two through forward and backward snowballing. Of the 40 remaining after duplicates were removed, we excluded 34 based on the title and abstract of the publications as they did not address our specific topic (e.g., not about tattooing or reported on tattoo prevalence or risk factors) or were in another language. Of the remaining six, five publications were deemed ineligible as they were just commentaries on safer tattooing projects, leaving one publication for a full-text review and qualitative synthesis. The publication retrieval process is detailed in Fig. 1 and the summary of reviewed studies in Table 3.

**Table 3:** Summary of studies included in the review

Study & context	Method or design	Sample or participants	Interventions	Health outcomes	Other outcomes	Study quality assessment
Nafekh et al. (2009) Correctional Services Canada 5 men’s & 1 women’s detention centers	Mixed-method: cross-sectional survey/questionnaire, interview with institutional staff members and incarcerated people	Men and women in detention	• 2-pronged: - *Operational*: a tattoo room in each center attended by trained detainee-tattooists. - *Educational:* information about the risks of unsafe tattooing for incarcerated people arriving at regional reception centers; guideline documents and pamphlets on the safer tattooing program for detained people from the pilot sites. • Supervision: by prison staff. • Duration: 7 months start-up, 12 months implementation, from August 2005 to August 2006. • Total costs: CAD 960′690. • Cost to client: CAD 5.00/2-h session. • Beneficiaries: 1043 tattooing sessions for 324 requests out of 384.	*• Enhanced level of knowledge and awareness amongst prison staff and incarcerated people regarding blood-borne infectious disease prevention and control practices.* • *Potential to reduce harm, reduce exposure to health risk, and enhance the health and safety of staff members (decreased injury from seizing illicit tattooing materials), incarcerated people and the general public with higher risk groups.* • *87% of interviewed people in detention would prefer to receive safer tattooing services.*	*• Additional employment opportunities for incarcerated people in the institution, and work skills that are transferable to the community.* • *Low cost respective to the benefit*. • *Perceived increase in the demand for tattoos.*	Weak

### Interventions

The included paper assessed a safer tattooing intervention in Canada in five men’s and a women’s detention centers [19]. The intervention started in 2005 and included an operational and an educational component. The operational component consisted of setting up a tattoo room in each of the six pilot centers. The tattoo room was designated as a controlled environment in meeting the standards for infection prevention and control. Safer tattooing was provided by detained individuals who became tattooists and after they had successfully completed training on BBIs and infection prevention and control practices. Supervision by prison staff ensured ongoing quality control of the services, as well as safety and security. In the educational component, all new individuals admitted to the regional reception centers received information about the risks of unsafe tattooing practices through educational materials, and the people already incarcerated received in the pilot sites a guidelines document and pamphlet on the safer tattooing program. Peer education and counseling was also integrated into the educational component.

### Methodological quality

The selected study was given a weak methodological rating. Although designed as a cross-sectional mixed-method evaluation at the end of the project, limitations that reduced the study quality included selection bias (moderate), study design (weak), confounders (weak), blinding (weak), and data collection (moderate).

### Health outcomes

The Canadian study suggests that their initiative resulted in an enhanced level of knowledge and awareness among staff and people in prison regarding BBI prevention and control practices. It appeared to demonstrate a potential to reduce harm, decrease exposure to health risk, and enhance the health and safety of staff members (decreased injury from seizing illicit tattooing materials), incarcerated people, and the general population. There was a reduction in illicit tattooing at medium security institutions, which was supported by the decrease in level of tattooing materials seized. Safer tattooing services was reportedly preferred by 87% of the interviewed detainees.

### Other outcomes

The Canadian project reportedly provided for participants additional employment opportunities and work skills, which could be transferable to the community. Beneficiaries and prison staff reported a perceived increase in the demand for tattoos. This may have been due to the low cost of tattooing. The project evaluators demonstrated that, as a harm reduction initiative, the project was overall of low cost when compared to the potential benefits, which included avoiding the direct costs of HCV treatment, HIV treatment, or liver transplant.

### Unpublished projects

The Luxemburg project started in April 2017 at the initiative of the prison health team who received the support from the carceral authorities. Based on evidence of clandestine tattooing in the prison, the project has the objective of providing a safer alternative to clandestine tattooing. Through a consultative process with the syndicate of people in prison, interventions were designed and consist of providing a tattoo parlor for trained tattooists to offer free-of-charge tattoos using safe tattooing materials and standards. A nurse (who is independent from the prison authorities) coordinates tattooing requests, manages tattooing materials, supervises tattooing sessions, and ensures that tattoo designs are acceptable (i.e., unrelated to violence, hate, gang, or radicalization). Privacy and confidentiality are ensured but prison staff knows the names of the individuals attending the tattooing sessions. There is no information, education, or counseling on tattooing risks for people who are incarcerated, except for tattooists. The program is available only to men who have been sentenced. The project is funded by the European Commission’s Erasmus+ program and reportedly running smoothly. There has been no published evaluation on its impact.

The project in Catalonia was not published and is only very briefly mentioned in an article overviewing harm-reduction strategies in detention centers in seven European countries [45]. Due to its focus on harm-reduction, the article was not identified in our search. However, the article was known by one of our co-authors. The tattoo-related harm-reduction intervention started in 2010 and consisted of providing safer tattooing information and making a professional tattooist available to people in prison. Tattoo restrictions, including of gang symbols, made the project unattractive – it was stopped within a year. There were no other details on intervention and outcomes.

## Discussion

Despite the evidence that tattooing is prevalent in prison settings, that BBIs are transmitted through unsafe tattooing, and safer tattooing practices are available to the general population and effective in preventing negative health consequences, this systematic review identified only one published research on safer tattooing from the grey literature. We found no peer-reviewed articles. Results from the Canadian project evaluation suggest that it was effective in enhancing knowledge of incarcerated people and prison staff on standard precautions, had the potential to reduce harm, provided vocational opportunities, and was feasible although enhancements were needed to improve implementation issues and efficiency. The project was halted due to the perceived low priority by the Government (“…taxpayer’s money should be put where it counts most. That means tackling crime, keeping drugs off our streets.”) [46]. The medical team of the Luxemburg prison is currently implementing a pilot project (still ongoing as of June 2018). Little is known about the closed Catalonian project.

The limited implementation of and research and publication on safer tattooing in prisons may be due to the clandestine and unregulated nature of tattooing in most closed settings. Making it licit and safer may receive limited support from criminal justice authorities, policymakers, and the public. In addition, policymakers may rely on the fact that there is no definitive evidence for a reduced risk of BBI transmission, especially HCV infection, when tattoos and piercings are done in professional parlors [24], although further evidence confirms that tattooing is an independent risk factor for HCV [27]. The public health importance of unsafe tattooing may be understandably overshadowed by injecting drug use in prisons, although there may be a higher proportion of people practicing unsafe tattooing than unsafe drug injection in prison settings [16, 17].

### Call to action for policy, practice, and research

Even though safer tattooing has been recommended by United Nations agencies as part of a comprehensive package for harm reduction in prisons, there is currently a dearth of evidence to inform the implementation of such practices. We call upon governments, criminal justice authorities, non-governmental organizations, and academic institutions to implement and evaluate safer tattooing projects. There is sufficient sound evidence to underpin the public health need for safer tattooing strategies. In addition, not offering such harm reduction intervention violates human rights law and international obligations to safeguard individuals who are sentenced to prison – they are not sentenced to be exposed to greater risk of BBIs while getting tattooed. People who experience incarceration keep their right to the highest standard of health while in detention, which includes the right to access preventive health and harm reduction services [47].

Implementation research is helpful to explore strategies to promote the systematic uptake of evidence-based practices, such as professional and safer tattooing services, which are available in the community [48]. Considering the scarce evidence regarding the implementation science on safer tattooing approaches, we call for more pilot initiatives on this neglected and understudied issue. These initiatives should adhere to the following guiding principles:

First, integration of methodologically-rigorous implementation research to help inform the decision-making of public health and custodial policymakers, managers, and practitioners. Safer tattooing techniques are available in the general population; how can they be implemented in carceral settings? Novel projects should therefore include robust implementation research to systematically document how proposed models are implemented and what operational barriers and enablers they encounter [49].

Second, involvement in the project design, implementation, and research of key stakeholders, including people who are incarcerated, public health and criminal justice authorities, prison and health staff, and research partners. This multi-stakeholder engagement is critical to conducting a participatory assessment of needs and resources, and to designing a project that is safe, acceptable, feasible, sustainable for all parties concerned, and potentially effective in demonstrating positive health outcomes. Sufficient time should be allocated to achieve the specific objectives of such project. For instance, demonstrating a reduction in BBI transmission, such as in the Canadian project, would require a long-term study with intervention and control groups – well beyond a year of implementation as it was done in that project.

**Fig. 2:**
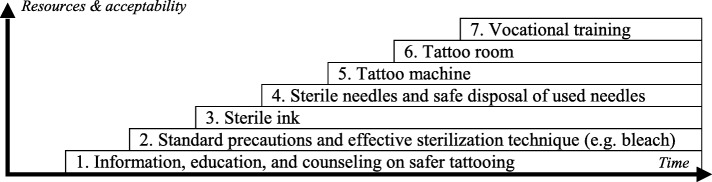
Model of a stepwise approach to safer tattooing in prison settings, according to time (x axis) and resources and acceptability from authorities (y axis)

Third, safer tattooing strategies in prisons should not operate in silos but be prioritized according to the public health needs and available resources in each detention setting and integrated within a comprehensive package of harm reduction strategies and BBI prevention, treatment, and care in prisons as defined by United Nations agencies [36]. In relation to safer tattooing, key interventions related to this comprehensive package of BBI management include for instance: information, education, and communication (IEC) on safer tattooing not only for tattoo recipients or tattooists but also for all incarcerated people and prison staff; BBI voluntary confidential counseling and testing, including for HIV, HCV, and HBV, and, if indicated, treatment, care, and support; standard precautions and effective sterilization techniques, such as bleach; sterile ink; single-use needles and safe disposal of used needles; tattoo machines; dedicated and supervised tattoo room; and vocational training. These interventions can be implemented in a stepwise approach according to available resources and acceptability thresholds from authorities (see Fig. 2). To have an impact on the BBI reservoir and modes of transmission, the other recommended interventions of the package should also be given prime importance, including the more contentious but effective needle and syringe exchange harm-reduction programs [50]. When designing safer tattooing projects, stakeholders should not miss the opportunity of integrating other forms of skin penetration, such as body piercing, which is a known risk factor for BBI transmission and which may be more prevalent among women [51].

Fourth, publication and dissemination of findings and, in case of positive outcomes, scaling up efforts must be a priority to help strengthen the body of evidence on safer tattooing in prisons and increase the alarmingly limited access to these harm-reduction practices for people who are incarcerated.

### Recommendations for further research

The lack of high quality evidence found in this review has led to the identification of several knowledge gaps. These gaps represent research opportunities to look at how to best implement and improve access to safer tattooing in prisons. Table 4 outlines recommendations for further research according to the five dimensions of accessibility as mentioned under Methods [44]. These recommendations will offer decision-makers much needed evidence on how to design, implement, and evaluate strategies to increase access to safer tattooing in detention settings. In terms of implementation research, the choice of study design will depend on the research question [49]. For instance, quantitative approaches may be considered to assess the extent to which information, education, and counseling materials influence knowledge, attitudes, and practices related to BBIs (e.g., pre- and post-intervention questionnaires); or to focus on the effectiveness of a selected harm reduction intervention for safer tattooing (e.g., pragmatic trial, or effectiveness-implementation hybrid trials [52], which have the advantage of assessing the effectiveness of both intervention and implementation strategy, and may be more relevant in prison settings and richer in information for decision-makers and program managers). The sensitivity around safer tattooing interventions may benefit from approaches that engage all key partners, including people who are incarcerated, in iterative processes of reflection, negotiation, and action. As such, participatory action research could be a suitable method as interventions would be implemented by concerned individuals for themselves rather done upon them – participant empowerment being an integral part of the process [53]. To further support, understand with nuances, and integrate multiple perspectives (e.g., insights into the willingness-to-pay for tattooing services) and multiple types of outcomes (e.g., acceptability, appropriateness, feasibility), mixed methods research may be considered as an approach to address a variety of implementation questions [54].

**Table 4:** Links between key areas of safer tattooing interventions, review findings, gaps in knowledge, and recommended focus for research

Key areas of safer tattooing interventions	Promising strategies based on findings	Gaps in knowledge	Areas for further research
Approachability (information provision)	Information and education materials given to all incarcerated people vs. only to tattooists and tattoo recipients.	Effect of information strategies over time on populations, including prison staff.	What information programs best increase awareness of both people in prison and prison staff and increase demand for safer tattooing services?
		Information on safer tattooing as part of an information package on blood-borne infection prevention, treatment, and care.	How to best integrate safer tattooing into a comprehensive infectious disease information package to increase demand for testing and counseling on HIV, HBV, HCV and other key infections?
			Are stand-alone information programs on unsafe tattooing risks as effective as providing information combined with safe tattoo room in reducing risks of blood-borne infection transmission through tattooing?
Acceptability	Supervision of a tattoo room by prison staff or by health staff.	Implementation of a tattoo room in prison health clinics (where such clinics are available) vs. in prison workshops.	Which implementation setting and supervision strategy are the most acceptable to people in prison, in addition to being cost-effective and feasible for the detention facility and the health services?
		Implementation of a stepwise model to safer tattooing that considers available resources and acceptability thresholds.	What is the effectiveness, feasibility, acceptability, and sustainability of each of the interventions outlined in a stepwise approach to safer tattooing (information, education and communication; standard precautions and effective sterilization techniques, such as bleach; sterile ink; single-use needles and safe disposal of used needles; tattoo machines; dedicated and supervised tattoo room; and vocational training)?
Availability of safer tattooing services	During non-working hours.	Availability limitations of the tattoo room when managed by health staff vs. prison staff.	How to best professionalize safer tattooing services into an official prison vocational workshop (thus guaranteeing quality services that are available during business hours)?
Affordability	Below-market costs or free-of-charge tattooing services.	Influence of direct costs borne by recipients on uptake of safer tattooing.	What is the willingness-to-pay of prospective recipients?
Appropriateness	Provision by people in prison trained to be tattooists.	Provision by trained detainee-tattooists vs. external professional tattooists vs. a combination of both?	What are the feasibility, sustainability, and acceptability of a private-public partnership between the detention center and private professional tattooist?
		Inclusion of other related services.	In addition to safer tattooing services, what are the other services to be offered, including non-health services (e.g. skin piercing), and health services (provision of health information on blood-borne infection prevention and screening)?

### Limitations

An incomplete retrieval of studies may have limited our research. However, efforts were made to hand search additional articles through backward and forward snowballing, include French and Spanish results in addition to English, and integrate grey literature. In addition, we asked stakeholders in Canada and Luxemburg to share their knowledge on other past or present projects around the world. The application of a narrative synthesis to the results of the reviewed study may have led to a loss of details, particularly of contextual factors that are important to the outcomes of the various interventions.

## Conclusions

While safer tattooing techniques are effective in preventing BBI transmission and available to the general population, this review identified only a few promising strategies to ensure access to safer tattooing interventions in detention settings. The guiding principles and research questions outlined in this article will help stakeholders take informed decision and action to avail safer tattooing interventions for people who experience incarceration. Such harm reduction and preventive measures will not only benefit people who receive and give tattoos in prisons but also the population at large.

## Additional file

Additional file 1: English translation of notes from in-depth interviews with nursing staff from the Luxemburg detention center. (DOCX 19 kb)

## Abbreviation

BBI: blood-borne infection
