# Supplementary material for: Safer tattooing interventions in prisons: a systematic review and call to action
Source: BMC Public Health. 2018 Aug 15;18:1015. doi: 10.1186/s12889-018-5867-x (PMC6094923; doi:10.1186/s12889-018-5867-x)
Supplement: Supplementary file 1 — English translation of notes from in-depth interviews with nursing staff from the Luxemburg detention center. (DOCX 19 kb) [file 12889_2018_5867_MOESM1_ESM.docx]

**Notes from in-depth interviews: tattoo room, Luxemburg detention center**

*Meeting on 28 June 17 with Jocelyne, nurse unit manager, Luxembourg prison. Further information obtained on 31 August 17 by telephone conversation with Mike, nurse in charge of the tattoo room.*

Ce projet a été rendu possible grâce au fonds européen Erasmus +, dans le cadre d’un programme d’éducation des pairs par les pairs. Au niveau de la chronologie, l’inscription au programme d’Erasmus + s’est faite en 2015 ; en avril 2016, les infirmiers ont reçu leur formation. La salle et le matériel ont été prêts dès avril 2017.

**Theme 1: Availability**

What was there before the establishment of the room?

There is a needle exchange program in the facility, as well as condom distribution.

Where did this project come from?

It was Sophie, a nurse from the unit who, following a personal interest, had launched the idea of this tattoo project. She had subsequently made it the theme of her dissertation work for a University Diploma in Public Health in Detention Setting.

The purpose of the project was not to remove illegal tattooing equipment, but to offer detainees something safer. The topic of tattoo risk reduction has emerged as a logical next step in a prison setting where a needle exchange program and condom distribution are already in place.

Have you contacted other facilities to help you set up?

No, there is no other prison in Luxembourg. We read the Canadian pilot project.

What are the access times to the room?

The room is available in the morning or afternoon and on weekends. However, the access to the room is more difficult during the weekend because of the limited availability of guards to accompany detainees to the tattoo room. During the week, this can be difficult because the detainee-tattoo-artist have other work to do.

The tattoo artists go zealously to the tattoo parlor, there is no absenteeism at this level.

The duration is maximum 4 hours per tattoo on a day, but there is no limit in the number of tattoos, nor in the time required for them.

Where is this room located?

The room sits within the medical service. Initially it was a storage room that was little used, and whose function was changed.

What is the role of the medical service in this structure? Who maintains the premises? Who is responsible for the hygiene, and the supply of the premises?

There are two nurses in charge of this project, and they are the ones who manage the whole process and are responsible for it.

These two nurses are responsible for the tattoo room, procurement, and the supervision of tattoos in terms of hygiene. They also ensure that there is no sign of radicalization. When an inmate wishes to have a tattoo, he or she asks for it via a letter addressed to the medical service. He can mention by which tattoo-artist he wishes to be tattooed. The inmate will then be received by the detainee-tattooist-artist and by one of the responsible nurses in order to define the project.

The tattoo room is available to detainees. The nurse can intervene in case of forbidden designs, but it will not intervene in case of discontent or disagreement between the detainees, who will have to find a solution between them. The role of the nurse is essentially to provide clean material.

**Theme 2: Acceptability**

With regard to confidentiality, human rights and privacy, how did you organize the room? Do the guards stay with you?

One of the responsible nurses is continuously present during tattooing and monitors hygiene conditions. The room is closed during the tattoo session. It is the responsible nurses who plan the tattooing sessions, and who are responsible for asking the prison authorities whether there are security issues between a detainee-tattooist and the inmate wishing to be tattooed.

With regard to confidentiality, the guards are not in the tattoo room, but know who comes to be tattooed because they manage the flow of patients in the medical service.

Within the medical team, how was this project received?

There was no noticeable concern. The topic of tattoo risk reduction has emerged as a logical next step in a prison setting where a needle exchange program and condom distribution are already in place.

Within the team, there are apparently two nurses who have a "problem" with the needle exchange program and also a little bit with the tattoo project, but they have been put back to their place by their superior.

As for the prison authorities, they disseminated the orders in a hierarchical way so that the project could not be debated or questioned.

**Theme 3: Appropriateness**

Does this project correspond to a real demand?

This project comes from an idea of the medical service, and especially from a nurse who was interested in the subject. She had met the "clandestine" tattooers before putting in place the project. It does not come from an idea / request from the management or the detainees.

Have you evaluated the program since its inception? how?

Program evaluation: completed questionnaire (by/for nurses) before and after the project started in the context of reporting to Erasmus+.

And the enabling factors?

Management was involved in the project and supported it from the start.

The two main problems reported by one of the nurses responsible for the room are:

- being "victims of their success" because they have a long waiting list. For the record, since April 2017 and the opening of the local, 60 tattoos have been made in 170 hours.

- the difficulty encountered in the accompaniment of detainees by the guards. This is all the more problematic as the time available for the tattoo room is already limited because of the working hours.

**Theme 4: Affordability**

Who paid for the room: the prison? the hospital? the inmates? all or part of it (only labor or material)?

This project was made possible by the European Erasmus+ fund, as part of a peer-to-peer education program. The Erasmus+ fund provided them with EUR 6000. The training of the nurses cost EUR1000, the two tattoo machines cost EUR400 each. Currently, they have EUR800 left and a lot of needles and ink because it is very cheap.

Access to the tattoo room is free for inmates.

This is a room in the medical service that has been refurbished for the project. This storage room was only rarely used, and its refurbishing was not expensive. As for the nursing staff, two nurses were assigned to this project, but there was no new position created: this was done with the existing staff.

As to whether this tattoo-artist work could become a vocational workshop as such, this is not possible. Indeed, in this prison, money exchange between two prisoners is forbidden. And bringing in an external tattoo-artist to make tattoos could increase the tattoo costs, and create inequality between people according to their economic resources.

**Theme 5: « Approachability »**

What was the mode of communication and information around this project? Have you done "advertising" for it?

There was no advertising or sign in the establishment. We met with the detainee syndicate once before starting the project, and then each syndicate representative disseminated the information to his quarter. There was no reminder from the medical service or verification of the information that had been transmitted between the detainees.

Was there a medical assessment prior to tattooing (psychiatric assessment, skin condition, serological assessment)? Did you take the opportunity to do prevention and information sessions around the risks of injections and/or tattooing?

There is no medical consultation or psychiatric evaluation before tattooing. As for the serologies, they are mandatory at the entrance, as well as a medical consultation and a chest x-ray. On the other hand, rapid tests for HIV are offered during the HIV day. Regarding the other prisoners in the prison, you should know that they all receive an information session on arrival on hepatitis and hygiene. This training is provided by the TOX (Communicable Disease Prevention) program, independent of the medical service.

In terms of information/prevention sessions, this was done for the detainee-tattoo-artists as part of their training.

Notes:

Initially, the nurses responsible for the tattoo project were accompanied by a doctor to go and to meet with the representatives of the detainee syndicate (one per quarter) to explain the project. Initially, the fear of the detainees was that this project was an attempt by the management to know who is a tattooing artist for control purposes. After clarification, they accepted because they understood that the goal was to provide them with something safer, and not to control or remove existing clandestine material.

Subsequently, those who wanted to become a tattooist had to write a letter of motivation addressed to the nurses. Once the tattoo artists had been selected, they received training on hygiene and blood-borne diseases.

In parallel, a professional tattoo artist met with the nurses, then met with the detainee-tattooist candidate to train on how to use the tattoo machine. Another private tattoo artist was contacted by the nurses and agreed to come and give half a day of training for the detainee-tattooist candidates. The main purpose of this training was to learn how the tattoo machine works, and how to handle the needles. He is not expected to return to train detainee-tattooists. Indeed, the initial goal of this project is peer-to-peer education. When there will be new tattoo artists to train, it will be the tattoo artists in place who will transfer knowledge to them.

The detainee-tattooists were trained by the nurses on hygiene and communicable diseases. There are no plans to refresh the knowledge of tattoo artists who were already trained.

In September 2017, new detainee tattooists will be trained and will receive training on hygiene and communicable diseases by nurses. The specific training for the tattoo machine will be done by the tattooist inmates themselves, as part of the peer-to-peer education program.

The initial goal of this project was to reduce the number of illegal tattooists. In the evaluation questionnaires, it was found that tattoo artists had a strong interest in tattooing, and that their interest in reducing risk was lower. Thus, they did not transmit information on this subject to other prisoners.

This work as a tattoo artist is not recognized outside, but according to the unit nurse, this can be an advantage. On the other hand, the rewarding aspect of being a tattoo artist and being recognized within the facility seems to be an important aspect. Tattoo artists have expressed their pride in being acknowledged and in tattooing with good material.

The room is available to all detainees, regardless of their criminal status. There has been no female candidate to be a tattoo artist, which is why they do not have access to the premises yet. Women can only be tattooed by women. It also seems that they demand more a room for piercing than tattooing.

The project was evaluated by questionnaires for tattooist and those who received tattoos in the context of Erasmus+ reporting.
